# Association of Dual Eligibility and Medicare Type With Quality of Postacute Care After Stroke

**DOI:** 10.1001/jamanetworkopen.2026.0095

**Published:** 2026-02-24

**Authors:** Amol M. Karmarkar, Lin-Na Chou, Tarang Jain, Robert Burke, Maricruz Rivera-Hernandez, Corey R. Fehnel, Margaret French, Amit Kumar

**Affiliations:** 1 Department of Physical Medicine and Rehabilitation, Center for Rehabilitation Science and Engineering, Virginia Commonwealth University, Richmond; 2 Clinical Science and Research Department, Sheltering Arms Institute, Richmond, Virginia; 3 Department of Physical Therapy & Athletic Training, University of Utah, Salt Lake City; 4 Department of Physical Therapy & Athletic Training, Idaho State University, Pocatello; 5 Division of General Internal Medicine, University of Pennsylvania Perelman School of Medicine, Philadelphia; 6 Leonard Davis Institute of Health Economics, University of Pennsylvania, Philadelphia; 7 Department of Health Services, Policy and Practice, Brown University, Providence, Rhode Island; 8 Center for Gerontology and Health Care Research, Brown University, Providence, Rhode Island; 9 Department of Neurology, Beth Israel Deaconess Medical Center, Harvard Medical School, Boston, Massachusetts; 10 Hinda and Arthur Marcus Institute for Aging Research, Boston, Massachusetts; 11 Center for Research on Race, Health Justice and Public Policy, University of Utah, Salt Lake City

## Abstract

**Importance:**

The growth of Medicare Advantage (MA) enrollment has reshaped postacute care utilization, particularly among dual-eligible beneficiaries who experience a disproportionate burden of stroke. Recent evidence shows that MA plans are proactive in managing care and directing enrollees toward narrower networks of postacute facilities for greater efficiency.

**Objective:**

To compare the likelihood of discharge to high-quality inpatient rehabilitation facilities (IRFs), skilled nursing facilities (SNFs), and home health (HH) care after stroke-related acute hospitalization among dual-eligible Medicare-Medicaid beneficiaries enrolled in MA vs Medicare fee-for-service (FFS) plans.

**Design, Setting, and Participants:**

This retrospective cohort study used a 20% random sample of Medicare data and included Medicare beneficiaries aged 65 years or older hospitalized for ischemic stroke between January 1, 2021, and September 3, 2022, with follow-up of postacute care use. The data were analyzed between February 1 and December 28, 2025.

**Exposure:**

Dual-eligible Medicare-Medicaid beneficiaries enrolled in FFS vs MA plans.

**Main Outcomes and Measures:**

Postacute care quality was assessed using the Centers for Medicare & Medicaid Services’ 5-star rating systems for SNFs and HH agencies. For IRFs, quality was defined by the rate of potentially preventable hospital readmissions during the IRF stay. All comparisons were risk adjusted for patient-, hospital-, and region-level factors.

**Results:**

In the cohort of 44 078 patients with stroke (mean [SD] age, 79.0 [8.3] years; 57.9% female), 20 497 (46.5%) were non–dual-eligible beneficiaries in FFS, 15 402 (34.9%) were non–dual-eligible beneficiaries in MA, 5256 (11.9%) were FFS dual-eligible beneficiaries, and 6190 (14.0%) were MA dual-eligible beneficiaries. Of the cohort, 17 350 (39.4%) were discharged to IRFs, 16 253 (36.9%) to SNFs, and 10 475 (23.8%) to HH care. There were no significant differences in the quality of IRFs used across groups. Compared with non–dual-eligible FFS beneficiaries, the likelihood of discharge to high-quality SNFs was lower for non–dual-eligible MA beneficiaries (odds ratio [OR], 0.82; 95% CI, 0.74-0.91), dual-eligible FFS beneficiaries (OR, 0.57; 95% CI, 0.50-0.65), and dual-eligible MA beneficiaries (OR, 0.56; 95% CI, 0.50-0.64). Similarly, non–dual-eligible MA beneficiaries were less likely to receive care from high-quality HH agencies (OR, 0.71; 95% CI, 0.62-0.82) compared with non–dual-eligible FFS beneficiaries.

**Conclusions and Relevance:**

In this cohort study, dual-eligible and MA-enrolled patients with stroke were less likely to receive postacute care from high-quality SNFs and HH agencies. Equitable access to high-value postacute care is essential to advancing outcomes for high-need, high-risk patients in the era of value-based care.

## Key Points

**Question:**

Are there differences by Medicare type and dual eligibility status associated with discharge to quality postacute care settings for patients with stroke?

**Findings:**

In this cohort study of 44 078 older patients hospitalized for stroke, dual-eligible beneficiaries in Medicare fee-for-service (FFS) plans, non–dual-eligible beneficiaries in Medicare Advantage (MA) plans, and dual-eligible beneficiaries in MA plans had a lower likelihood of discharge to high-quality nursing homes compared with non–dual-eligible beneficiaries in FFS Medicare plans.

**Meaning:**

This study suggests that dual-eligible and MA beneficiaries with stroke experience disparities in receiving high-quality postacute care; improving awareness of postacute care facility quality ratings among patients, caregivers, and discharge planners, along with having high-quality facilities in the MA plan network, can help reduce these disparities.

## Teaser

This cohort study compares the likelihood of discharge to high-quality postacute care settings after stroke-related acute hospitalization among dual-eligible Medicare-Medicaid beneficiaries enrolled in Medicare Advantage vs Medicare fee-for-service plans.

## Introduction

After acute hospitalization for stroke, patients often experience significant disability and require high-quality postacute care (PAC) to optimize functional recovery.^1^ Stroke-related PAC is typically provided through inpatient rehabilitation facilities (IRFs), skilled nursing facilities (SNFs), or home health (HH) services.^2^ Nevertheless, the PAC in the US is characterized by fragmentation based on intensity of care and payment structures.^3^ As a result, considerable variability has been reported in both discharge patterns and health outcomes—such as community discharge, hospital readmission, and mortality—across these settings.^4,5,6,7,8,9,10,11^ However, variability in the quality of care within PAC settings has not been fully explored among patients with stroke. Existing research on PAC utilization after stroke has focused primarily on Medicare fee-for-service (FFS) beneficiaries, overlooking the rapidly growing number of beneficiaries with Medicare Advantage (MA) plans, which now account for approximately 54% of all Medicare beneficiaries.^12^

One major factor associated with PAC services is the insurance coverage and allowance for the services. Unlike the traditional FFS plans, MA plans use a narrow network of providers and facilities, prior authorization requirements, cost sharing, and other strategies to manage (control) utilization of PAC.^8,13,14,15,16,17,18,19,20^ These strategies may influence the discharge planning process and decisions regarding PAC discharge destinations. Inadvertently, these strategies also result in individuals on MA plans facing a different choice set of PAC settings or differential quality of PAC settings or substitution of PAC settings.^7,8^ In addition, dual-eligible beneficiaries enrolled in MA plans often face care coordination challenges,^21^ particularly with PAC, and may receive different levels or quality of clinical care compared with dual-eligible beneficiaries enrolled in FFS plans. Given the critical role of PAC in stroke recovery, it is important to understand how high-need dual-eligible patients with stroke enrolled in MA vs FFS programs experience differences in the quality of care across PAC settings.

Individuals who qualify for both Medicare and Medicaid programs, considered dual eligible, are a particularly important group of individuals to examine because they face a disproportionate stroke burden and greater stroke severity.^22^ Prior evidence shows that dual-eligible beneficiaries have a lower likelihood of access and utilization of high-quality SNFs, which was negatively associated with health outcomes (eg, hospital readmissions).^23,24,25^ The dual-eligible populations now constitute more than 50% of enrollees in MA plans and face doubled challenges after stroke hospitalization due to the higher stroke severity and disease burden as well as restricted access to PAC facilities due to MA plans’ restrictions. For patients with stroke, the transition from acute care to PAC is a critical and brief period. This period poses challenges for dual-eligible beneficiaries in MA plans, who may encounter barriers such as limited provider networks and prior authorization requirements. To date, no studies have examined how MA enrollment and dual eligibility together are associated with PAC quality after stroke. As the populations of MA enrollees and dual-eligible beneficiaries continue to grow, understanding this intersection is important to improving the quality of poststroke care for high-need beneficiaries. Therefore, this study evaluates differences in the quality of IRFs, SNFs, and HH services used by MA and FFS beneficiaries after stroke, with a focus on high-needs dual-eligible populations.

## Methods

The study was approved by the institutional review board at the University of Utah with a waiver of informed consent due to use of secondary deidentified data via a data use agreement with the Centers for Medicare & Medicaid Services (CMS). We followed the Strengthening the Reporting of Observational Studies in Epidemiology (STROBE) reporting guideline.^26^

### Study Design and Data Sources

We used a 20% sample of patient-level Medicare claims data, including the Master Beneficiary Summary File (MBSF), Medicare Provider Analysis and Review (MedPAR) file, and 3 postacute assessment files from January 1, 2021, to September 30, 2022. The MBSF contains information on beneficiaries’ sociodemographic characteristics, as well as indicators for Medicare enrollment such as MA, FFS, and dual-eligible status. The MedPAR file contains all the claims information related to acute hospitalization. The postacute assessment files included the Inpatient Rehabilitation Facility Patient Assessment Instrument (IRF-PAI) for IRFs, Minimum Data Set (MDS-3.0) for SNFs, and the Outcome and Assessment Information Set (OASIS) for HH care. These postacute assessments are completed for all admissions and discharges during stays in these settings, regardless of the patient’s insurance status (MA and FFS). We obtained quality of care ratings information from the Medicare Care Compare^27^ for IRFs, SNFs, and HH agencies. For SNFs and HH agencies, we used the composite 5-star rating systems. For IRFs, we used one of the CMS quality measures—rate of potentially preventable hospital readmissions during the IRF stay—to assess quality rating. We obtained the facility and national rate of potentially preventable hospital readmissions during the IRF stay from 2021, aligning with the study cohort period. The Provider of Services file was used to retrieve information on hospital-level data such as profit status and teaching status. We used publicly available data on county-level MA penetration retrieved from the CMS website. MA penetration is calculated by dividing the number of beneficiaries enrolled in MA plans by the number of eligible Medicare beneficiaries in a county for a given month.

### Study Sample Population

**Figure 1.**
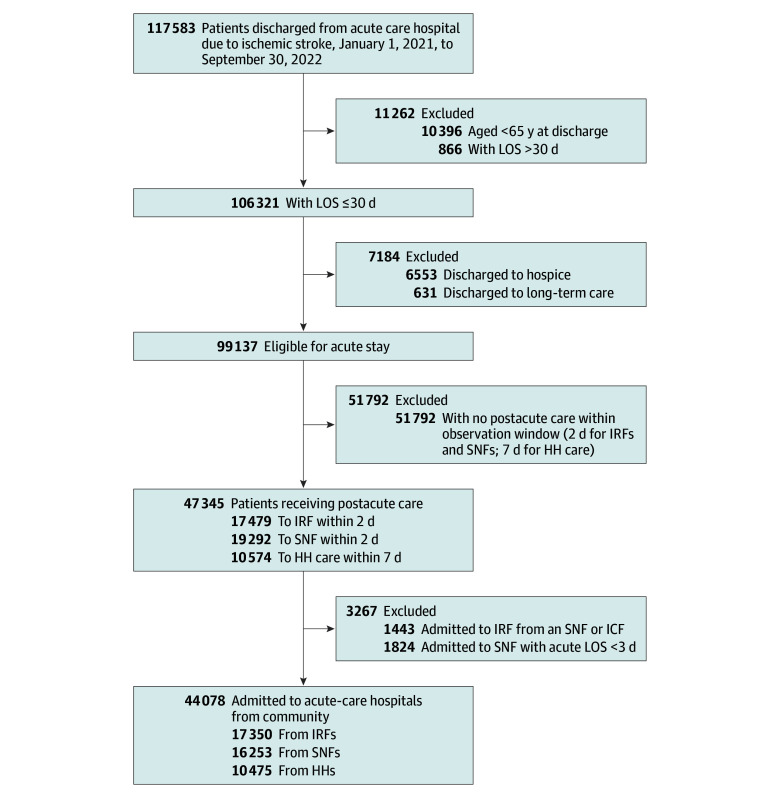
Flowchart of Study Cohort Selection HH indicates home health; ICF, intermediate care facility; IRF, inpatient rehabilitation facility; LOS, length of stay; and SNF, skilled nursing facility.

The cohort included Medicare beneficiaries aged 65 years or older enrolled either in FFS or MA plans, who were admitted to acute-care hospitals between January 1, 2021, and September 30, 2022, with ischemic stroke. The diagnosis of ischemic stroke was identified using the primary *International Statistical Classification of Diseases and Related Health Problems, Tenth Revision, Clinical Modification* (*ICD-10-CM*), code (I63.x and I67.8). We included patients discharged from acute-care hospitals followed by direct admission (operationally defined as within 48 hours) to IRFs or SNFs. Patients discharged to home were included based on receipt of HH services within 7 days of acute-care hospital discharge. Postacute admissions were determined using the subsequent claims and assessments information from IRF-PAI, MDS-3.0, and OASIS. These time frames were selected using the previous literature as a guideline^16,28,29,30^ and to minimize acute to PAC transition time. We excluded patients with an acute-care hospitalization length of stay greater than 30 days, those readmitted from SNFs or intermediate care facilities, and those discharged to hospice or long-term care. The final analytic sample included 44 078 patients with ischemic stroke discharged to IRFs, SNFs, or HH settings after acute hospitalization (Figure 1).

### Primary Variables of Interest

The primary independent variables were (1) MA vs FFS plans and (2) dual-eligible status (dual vs non–dual eligible), derived from the enrollment status in the Master Beneficiary Summary File (MBSF). The MBSF contains data on beneficiary characteristics and monthly enrollment in Part A (inpatient), Part B (outpatient), Part C (MA, managed care, or health maintenance organization), and Part D (prescription drug coverage) of all Medicare beneficiaries enrolled in or entitled to Medicare within a given calendar year. Beneficiaries were classified as MA or FFS based on their enrollment status in the MBSF during the time of stroke hospitalization (month of stroke-related hospitalization). Likewise, dual-eligibility status was obtained from the MBSF, which provides monthly Medicaid eligibility information. In our study, patients were classified as dual eligible if they were enrolled in both Medicare and Medicaid at the time of stroke hospitalization and during their PAC stay. We created 4 mutually exclusive categories: (1) FFS non–dual eligible, (2) FFS dual eligible, (3) MA non–dual eligible, and (4) MA dual eligible.

### Outcomes

We used 5-star ratings at the SNF level, categorizing SNFs with star ratings of 1 to 3 as low quality and those with star ratings of 4 to 5 stars as high quality.^31,32,33^ We classified HH care agencies into low quality (1-4 stars) and high quality (>4 stars).^34^ The CMS does not publish star ratings for IRFs; therefore, as established by the CMS and used by prior research, we used the rate of potentially preventable hospital readmissions during the IRF stay to assess quality.^35,36^ We classified an IRF as low quality if the rate of potentially preventable hospital readmissions during the IRF stay for that particular IRF was higher than the national mean.^37^

### Covariates

To account for case mix and other variables, we included patient-level demographic and clinical characteristics in our analysis. The demographic characteristics include age, sex, race and ethnicity (Asian or Pacific Islander, Hispanic, non-Hispanic Black, non-Hispanic White, and other [American Indian or Alaska Native, unknown, and other]), using Research Triangle Institute race codes from MBSF files. We also included patient residence location (urban vs rural, defined by 2023 Rural-Urban Continuum Codes), resident of a stroke belt state (Alabama, Arkansas, Georgia, Indiana, Kentucky, Louisiana, Mississippi, North Carolina, South Carolina, Tennessee, and Virginia),^38^ and county-level MA penetration rate. The 2023 Rural-Urban Continuum Codes distinguish US metropolitan counties by the population size of their metropolitan area, and nonmetropolitan counties by their degree of urbanization and adjacency to a metropolitan area. We labeled the metropolitan counties as urban and nonmetropolitan counties as rural. To account for clinical characteristics, we included lengths of stay during acute hospitalization, intensive care unit stay, receipts of tissue plasminogen activator (tPA), claims-based National Institutes of Health Stroke Scale (NIHSS), and Charlson Comorbidity Index from hospital claims. Using claims-based and validated cutoff points, stroke severity scale (NIHSS) was combined and categorized as mild (0-9), moderate to severe (10-42), or unknown.^22,39^ The unknown category included patients with missing NIHSS data. We identified tPA by reviewing all 25 *International Statistical Classification of Diseases and Related Health Problems, Tenth Revision* (*ICD-10*), procedure codes reported in the MedPAR files and flagging records containing the relevant codes (3E03317, 3E04317, 3E05317, and 3E06317). For NIHSS we used *ICD-10-CM* codes from the MedPAR files that were validated previously.^22,39^ We assessed whether patients received rehabilitation services during their acute care, reflecting the quality of care provided during their hospital stay.^40,41^ Hospital-level variables included profit status, teaching status, and volume of patients with stroke treated annually. At the geographic level, we included the number of MA plans available within each Hospital Referral Region (HRR) to account for MA penetration, as well as the availability of PAC facilities, including IRFs, SNFs, and HH agencies. We used publicly available data on county-level MA penetration by month retrieved from the CMS website. MA penetration is calculated by dividing the number of beneficiaries enrolled in MA plans by the number of eligible Medicare beneficiaries in a county for a given month. For PAC availability, we linked 2021 Medicare Provider Data with zip code–to–HRR crosswalk to calculate the number of IRFs, SNFs, and HH agencies within each HRR. For all 3 PAC settings, we included admission functional assessment scores in the self-care and mobility domains using the standardized Section GG measures from the Continuity Assessment Record and Evaluation tool.^42,43^

### Statistical Analysis

Statistical analysis was conducted from February 1 to December 28, 2025. Descriptive statistics were used to summarize patient demographic characteristics, clinical characteristics, hospital attributes, and geographic factors, stratified by 4 mutually exclusive groups: FFS non–dual eligible, FFS dual eligible, MA non–dual eligible, and MA dual eligible. We also examined the main association of insurance status (FFS vs MA plans) and dual eligibility (dual vs non–dual eligible) and the interaction between insurance status and dual eligibility (eTable 2 in Supplement 1). We used χ^2^ tests for categorical variables and analysis of variance for continuous variables to examine the associations between covariates and the 4 groups. Continuous variables are reported with mean (SD) values, while categorical variables are presented with frequencies and percentages in Table 1. In addition, we presented stroke severity, admission functional scores (self-care and mobility status), and PAC facility characteristics (quality ratings and types of ownership), stratified by 3 PAC settings. We analyzed the association between the 4 mutually exclusive groups (FFS non–dual eligible [reference group], FFS dual eligible, MA non–dual eligible, and MA dual eligible) and the quality of PAC services. We adjusted for patient-, hospital-, and HRR-level covariates and accounted for the nested structure of the data, addressing the clustering at the hospital and HRR in multilevel logistic models. A 3-level hierarchical structure was specified, with patients nested within hospitals and hospitals nested in HRR. The models were fitted using the Laplace approximation for maximum likelihood estimation. Two random intercepts were included to account for clustering at higher levels: one at the HRR level and one at the hospital level, each assuming a variance-components covariance structure. The fixed-effects portion of the model included the individual-, hospital-, and HRR-level covariates. All tests of statistical significance were 2-sided, *P* < .05 was considered statistically significant for adjusted analyses, and all analyses were performed with SAS, version 9.4 (SAS Institute Inc).

**Table 1.** Descriptive Characteristics of Mutually Exclusive Groups of Patients After Hospital Discharge for Ischemic Stroke (N = 44 078)

Variable	Medicare FFS		MA	
	Non–dual eligible	Dual eligible	Non–dual eligible	Dual eligible
Patient level				
No. of patients	20 497	5256	15 402	6190
Age, mean (SD), y^a^^,^^b^^,^^c^^,^^d^	80.0 (8.2)	77.9 (8.8)	79.2 (8.0)	76.5 (8.2)
Sex, No. (%)^a^^,^^b^^,^^d^				
Male	8403 (43.8)	1706 (37.8)	6404 (43.6)	2053 (36.0)
Female	10 794 (56.2)	2802 (62.2)	8270 (56.4)	3646 (64.0)
Race and ethnicity, No. (%)^a^^,^^b^^,^^c^^,^^d^				
Asian or Pacific Islander	292 (1.5)	367 (8.1)	267 (1.8)	229 (4.0)
Hispanic	581 (3.0)	572 (12.7)	955 (6.5)	890 (15.6)
Non-Hispanic Black	1797 (9.4)	978 (21.7)	2229 (15.2)	1870 (32.8)
Non-Hispanic White	16 125 (84.0)	2462 (54.6)	10 982 (74.8)	2605 (45.7)
Other^e^	402 (2.1)	129 (2.9)	241 (1.6)	105 (1.8)
Residential area, No. (%)^a^^,^^c^^,^^d^				
Urban	15 335 (80.6)	3500 (79.0)	12 592 (86.8)	4728 (84.9)
Rural	3690 (19.4)	930 (21.0)	1920 (13.2)	840 (15.1)
Resident in stroke belt, No. (%)^a^^,^^b^^,^^c^				
Yes (11 states)	5154 (26.8)	1012 (22.4)	3313 (22.6)	1527 (26.8)
No	14 043 (73.2)	3496 (77.6)	11 361 (77.4)	4172 (73.2)
MA penetration (county of residence), No. (%)^a^^,^^b^^,^^c^^,^^d^				
≤National mean (42% in 2021)	10 068 (52.5)	2021 (44.9)	4448 (30.3)	1797 (31.6)
>National mean (42% in 2021)	9110 (47.5)	2476 (55.1)	10 211 (69.7)	3897 (68.4)
Charlson Comorbidity Index, mean (SD)^a^^,^^b^^,^^c^^,^^d^	3.1 (1.4)	3.4 (1.4)	3.2 (1.4)	3.5 (1.4)
LOS in acute hospital, mean (SD), d^a^^,^^b^^,^^c^^,^^d^	5.4 (4.2)	6.4 (4.9)	6.7 (4.8)	7.7 (5.4)
ICU stay, No. (%)^a^^,^^c^				
No	10 418 (54.3)	2449 (54.3)	7665 (52.2)	3032 (53.2)
Yes	8779 (45.7)	2059 (45.7)	7009 (47.8)	2667 (46.8)
NIHSS, No. (%)^a^^,^^b^^,^^c^^,^^d^				
Score 0-9 (mild)	9781 (51.0)	1754 (38.9)	7397 (50.4)	2534 (44.5)
Score 10-42 (moderate to severe)	2871 (15.0)	908 (20.1)	2423 (16.5)	1101 (19.3)
Unknown	6545 (34.1)	1846 (40.9)	4854 (33.1)	2064 (36.2)
Intravenous tPA during the inpatient stay, No. (%)^a^^,^^b^	1769 (9.2)	346 (7.7)	1443 (9.8)	498 (8.7)
Hospital-based rehabilitation, No. (%)^a^^,^^b^^,^^c^^,^^d^	19 029 (99.1)	4418 (98.0)	14 242 (97.1)	5586 (98.0)
Postacute setting, No. (%)^a^^,^^b^^,^^c^^,^^d^				
IRF within 2 d	8978 (46.8)	1282 (28.4)	5576 (38.0)	1514 (26.6)
SNF within 2 d	5686 (29.6)	2309 (51.2)	5345 (36.4)	2913 (51.1)
HH agency within 7 d	4533 (23.6)	917 (20.3)	3753 (25.6)	1272 (22.3)
Hospital level (discharging hospital)				
No. of hospitals	2438	1638	2196	1636
Profit status, No. (%)				
Profit	425 (17.4)	273 (16.7)	381 (17.3)	285 (17.4)
Nonprofit or government	2013 (82.6)	1365 (83.3)	1815 (82.7)	1351 (82.6)
Teaching status, No. (%)^a^^,^^b^^,^^d^				
Major and limited	940 (38.6)	748 (45.7)	909 (41.4)	746 (45.6)
No affiliation	1498 (61.4)	890 (54.3)	1287 (58.6)	890 (54.4)
Hospital stroke volume, No. (%)^a^^,^^b^^,^^c^^,^^d^				
Low (<45 ischemic stroke discharges)	1355 (55.6)	715 (43.7)	1117 (50.9)	684 (41.8)
High (≥45 ischemic stroke discharges)	1083 (44.4)	923 (56.3)	1079 (49.1)	952 (58.2)
Geographic level (HRR)				
No. of HRRs	307	300	307	296
No. of MA plans in HRR, mean (SD)	122.8 (64.0)	123.7 (64.3)	122.8 (64.0)	125.3 (63.7)
No. of facilities or agencies in HRR, mean (SD)				
IRF	3.9 (4.2)	4.0 (4.2)	3.9 (4.2)	4.0 (4.2)
SNF	49.8 (48.6)	50.4 (48.9)	49.8 (48.6)	50.9 (49.1)
HH agency	36.4 (78.8)	36.9 (79.6)	36.4 (78.8)	37.4 (80.1)

Abbreviations: FFS, fee-for-service; HH, home health; HRR, hospital referral region; ICU, intensive care unit; IRF, inpatient rehabilitation facility; LOS, length of stay; MA, Medicare Advantage; NIHSS, National Institutes of Health Stroke Scale; SNF, skilled nursing facility; tPA, tissue plasminogen activator.

^a^ *P* < .01 for 4-group comparison.

^b^ *P* < .01 for group comparison between FFS non–dual and FFS dual eligible.

^c^ *P* < .01 for group comparison between FFS non–dual and MA non–dual eligible.

^d^ *P* < .01 for group comparison between FFS non–dual and MA dual eligible.

^e^ Other category (n = 877) comprises Research Triangle Institute race codes classified as American Indian or Alaska Native (n = 162), other (n = 348), and unknown (n = 367).

## Results

### Descriptive Results of Postacute Care Discharge Patterns

In this cohort of 44 078 patients with stroke, the 4 mutually exclusive groups for the study were FFS non–dual eligible (20 497 [46.5%]; mean [SD] age, 80.0 [8.2] years; 10 794 women [56.2%] and 8403 men [43.8%]; 292 Asian or Pacific Islander [1.5%], 581 Hispanic [3.0%], 1797 non-Hispanic Black [9.4%], 16 125 non-Hispanic White [84.0%], and 402 other race or ethnicity [2.1%]), FFS dual eligible (5256 [11.9%]; mean [SD] age, 77.9 [8.8] years; 2802 women [62.2%] and 1706 men [37.8%]; 367 Asian or Pacific Islander [8.1%], 572 Hispanic [12.7%], 978 non-Hispanic Black [21.7%], 2462 non-Hispanic White [54.6%], and 129 other race or ethnicity [2.9%]), MA non–dual eligible (15 402 [34.9%]; mean [SD] age, 79.2 [8.0] years; 8270 women [56.4%] and 6404 men [43.6%]; 267 Asian or Pacific Islander [1.8%], 955 Hispanic [6.5%], 2229 non-Hispanic Black [15.2%], 10 982 non-Hispanic White [74.8%], and 241 other race or ethnicity [1.6%]), and MA dual eligible (6190 [14.0%]; mean [SD] age, 76.5 [8.2] years; 3646 women [64.0%] and 2053 men [36.0%]; 229 Asian or Pacific Islander [4.0%], 890 Hispanic [15.6%], 1870 non-Hispanic Black [32.8%], 2605 non-Hispanic White [45.7%], and 105 other race or ethnicity [1.8%]). Table 1 provides individual-, hospital-, and HRR-level characteristics and differences between these groups. There were 17 350 patients with stroke (39.4%) discharged to IRFs, 16 253 (36.9%) to SNFs, and 10 475 (23.8%) to HH care (Figure 1). Table 2 presents the descriptive characteristics of the 4 mutually exclusive beneficiary groups across the 3 PAC settings. For patients discharged to IRFs, we found statistically significant differences for stroke severity, admission functional status, and the quality of IRF among the 4 groups. For the quality of IRF, 767 of 1514 MA dual-eligible patients (52.5%) were discharged to low-quality IRFs compared with 3965 of 8978 FFS non–dual-eligible patients (46.6%). Across the 4 groups, 6133 of 17 350 patients (38.3%) were admitted to for-profit IRFs, with no significant differences observed among groups. Among those discharged to SNFs, statistically significant differences were observed in stroke severity, admission functional status, facility ownership, and facility quality ratings across the 4 groups. For the quality of SNF, 1425 of 2913 MA dual-eligible patients (58.3%) were discharged to low-quality SNFs, compared with 2092 of 5686 FFS non–dual-eligible patients (42.0%). In addition, 1881 of 2913 MA dual-eligible patients (76.1%) received care in for-profit SNFs compared with 3391 of 5686 FFS non–dual-eligible patients (67.1%). For patients discharged to HH care, we found statistically significant differences for stroke severity, admission functional status, facility ownership, and the quality of facility among the 4 groups. For the quality of HH agencies, 964 of 1272 MA dual-eligible patients (76.3%) were discharged to low-quality HH agencies, compared with 3496 of 4533 FFS non–dual-eligible patients (78.1%). In addition, 736 of 1272 MA dual-eligible patients (58.1%) received care from for-profit HH agencies, compared with 2351 of 4533 FFS non–dual-eligible patients (52.0%).

**Table 2.** Characteristics and Quality of Postacute Care Settings Among 4 Mutually Exclusive Groups of Patients After Hospital Discharge for Ischemic Stroke

Variable	Medicare FFS		MA	
	Non–dual eligible	Dual eligible	Non–dual eligible	Dual eligible
**Inpatient rehabilitation facility (n = 17 350)**				
No. of patients	8978	1282	5576	1514
NIHSS, No. (%)^a^^,^^b^^,^^c^				
Score 0-9 (mild)	4831 (53.8)	605 (47.2)	2985 (53.5)	773 (51.1)
Score 10-42 (moderate to severe)	1549 (17.3)	251 (19.6)	996 (17.9)	316 (20.9)
Unknown	2598 (28.9)	426 (33.2)	1595 (28.6)	425 (28.1)
Functional score at admission				
Self-care (7 items), mean (SD)^a^^,^^b^^,^^c^	20.0 (6.7)	19.0 (6.5)	19.9 (6.5)	19.2 (6.4)
Mobility (15 items), mean (SD)^a^^,^^b^^,^^c^	33.1 (13.4)	31.3 (12.3)	32.7 (12.9)	31.4 (12.4)
Quality of facility, No. (%)^a^^,^^c^^,^^d^				
Low (readmission during stay ≥ national mean)	3965 (46.6)	615 (50.1)	2564 (47.8)	767 (52.5)
High (readmission rate during stay < national mean)	4537 (53.4)	612 (49.9)	2800 (52.2)	695 (47.5)
Facility ownership^d^				
Profit	3202 (38.9)	451 (38.6)	1947 (37.5)	533 (38.1)
Nonprofit or government	5028 (61.1)	716 (61.4)	3251 (62.5)	866 (61.9)
**Skilled nursing facility (n = 16 253)**				
No. of patients	5686	2309	5345	2913
NIHSS, No. (%)^a^^,^^b^^,^^c^^,^^e^				
Score 0-9 (mild)	2338 (41.1)	707 (30.6)	2320 (43.4)	1057 (36.3)
Score 10-42 (moderate to severe)	1055 (18.6)	552 (23.9)	1082 (20.2)	673 (23.1)
Unknown	2293 (40.3)	1050 (45.5)	1943 (36.4)	1183 (40.6)
Functional score at admission				
Self-care (7 items), mean (SD)^a^^,^^b^^,^^c^^,^^e^	18.2 (7.7)	16.1 (7.8)	12.4 (7.6)	12.4 (7.6)
Mobility (15 items), mean (SD)^a^^,^^b^^,^^c^^,^^e^	27.6 (11.7)	24.9 (11.2)	21.0 (10.0)	20.7 (9.7)
Quality of facility, No. (%)^a^^,^^b^^,^^c^^,^^e^^,^^f^				
Low (overall score 1-3)	2092 (42.0)	1075 (56.2)	2172 (46.8)	1425 (58.3)
High (overall score 4-5)	2884 (58.0)	839 (43.8)	2473 (53.2)	1021 (41.7)
Facility ownership^a^^,^^b^^,^^c^^,^^e^^,^^f^				
Profit	3391 (67.1)	1516 (77.9)	3326 (70.9)	1881 (76.1)
Nonprofit or government	1666 (32.9)	431 (22.1)	1367 (29.1)	591 (23.9)
**Home health agency (n = 10 475)**				
No. of patients	4533	917	3753	1272
NIHSS, No. (%)^a^^,^^b^^,^^c^^,^^e^				
Score 0-9 (mild)	2612 (57.6)	442 (48.2)	2092 (55.7)	704 (55.3)
Score 10-42 (moderate to severe)	267 (5.9)	105 (11.5)	345 (9.2)	112 (8.8)
Unknown	1654 (36.5)	370 (40.3)	1316 (35.1)	456 (35.8)
Functional score at admission				
Self-care (7 items), mean (SD)^a^^,^^b^^,^^c^	25.2 (6.9)	21.9 (7.9)	25.6 (7.2)	23.8 (7.7)
Mobility (15 items), mean (SD)^a^^,^^b^^,^^c^	44.5 (14.3)	38.6 (15.5)	44.6 (14.6)	41.5 (15.3)
Quality of facility, No. (%)^a^^,^^d^^,^^e^				
Low (overall score 1-4)	3496 (78.1)	712 (79.7)	3039 (81.4)	964 (76.3)
High (overall score 4.5-5)	982 (21.9)	181 (20.3)	696 (18.6)	299 (23.7)
Facility ownership^a^^,^^b^^,^^c^^,^^d^^,^^e^				
Profit	2351 (52.0)	538 (59.1)	1810 (48.3)	736 (58.1)
Nonprofit or government	2166 (48.0)	372 (40.9)	1937 (51.7)	530 (41.9)

Abbreviations: FFS, fee-for-service; MA, Medicare Advantage; NIHSS, National Institutes of Health Stroke Scale.

^a^ *P* < .01 for 4-group comparison.

^b^ *P* < .01 for group comparison between FFS non–dual and FFS dual eligible.

^c^ *P* < .01 for group comparison between FFS non–dual and MA dual eligible.

^d^ There are missing data.

^e^ *P* < .01 for group comparison between FFS non–dual and MA non–dual eligible.

^f^ More than 10% missing data.

### Quality of PAC and Associated Multilevel Factors

Table 3 presents the results from multilevel logistic regression models examining the association between the 4 mutually exclusive groups and the likelihood of discharge to high-quality PAC. We have provided all the adjusted odds ratios (ORs) associated with all the covariates in eTable 1 in Supplement 1. The analyses were stratified by IRF, SNF, and HH care, with a separate model estimated for each setting. For all models, the FFS non–dual-eligible group served as the reference category. All analyses were risk adjusted for individual-, hospital-, and HRR-level factors. For IRFs, we did not find any significant association for discharge to high-quality IRFs with the 4 groups. For SNFs, compared with the FFS non–dual-eligible group, the likelihood of discharge to a high-quality facility was lower among FFS dual-eligible patients (OR, 0.57; 95% CI, 0.50-0.65), MA non–dual-eligible patients (OR, 0.82; 95% CI, 0.74-0.91), and MA dual-eligible patients (OR, 0.56; 95% CI, 0.50-0.64) (Table 3). For HH agencies, compared with the FFS non–dual-eligible group, MA non–dual-eligible patients had a lower likelihood of discharge to a high-quality HH agency (OR, 0.71; 95% CI, 0.62-0.82). For the HRR-level factor, a higher number of SNFs in a given HRR was associated with a lower likelihood of discharge to a higher-quality HH agency (OR, 0.56; 95% CI, 0.35-0.88), whereas a higher concentration of MA plans within an HRR was associated with a greater likelihood of discharge to high-quality SNFs (OR, 1.45; 95% CI, 1.17-1.81).

**Table 3.** Association of Medicare Plan Type and Dual-Eligible Status With Receipt of High-Quality Postacute Care

Variable^a^	Odds ratio (95% CI)^b^		
	IRF	SNF	HH agency
No.	16 180	10 137	10 283
Patient level			
FFS non–dual eligible	1.00 [Reference]	1.00 [Reference]	1.00 [Reference]
FFS dual eligible	0.89 (0.70-1.14)	0.57 (0.50-0.65)^c^	0.79 (0.62-1.01)
MA non–dual eligible	0.98 (0.85-1.13)	0.82 (0.74-0.91)^c^	0.71 (0.62-0.82)^c^
MA dual eligible	1.12 (0.89-1.40)	0.56 (0.50-0.64)^c^	0.91 (0.74-1.13)
Hospital level			
Major teaching affiliation	0.85 (0.56-1.27)	1.06 (0.94-1.20)	0.86 (0.71-1.05)
High volume of strokes	0.96 (0.63-1.45)	1.14 (1.01-1.29)^c^	0.96 (0.78-1.18)
HRR level			
High No. of MA plans	0.94 (0.52-1.71)	1.45 (1.17-1.81)^c^	1.10 (0.73-1.66)
High No. of IRFs	1.09 (0.57-2.10)	1.02 (0.80-1.29)	1.18 (0.74-1.85)
High No. of SNFs	1.42 (0.74-2.72)	0.91 (0.72-1.16)	0.56 (0.35-0.88)^c^
High No. of HH agencies	0.55 (0.29-1.05)	0.82 (0.64-1.04)	1.25 (0.79-1.97)

Abbreviations: FFS, fee-for-service; HH, home health; HRR, hospital referral region; IRF, inpatient rehabilitation facility; MA, Medicare Advantage; SNF, skilled nursing facility.

^a^ Only patients with complete data are included in the analyses.

^b^ Multilevel logistic regression model (HRR, hospital, and patient) included the adjustment of patient-level covariates (age, sex, race and ethnicity, metropolitan residential area, stroke belt location, MA penetration, Charlson Comorbidity Index, acute hospital length of stay, intensive care unit stay, National Institutes of Health Stroke Scale score, intravenous thrombolytic, inpatient rehabilitation, admission self-care score, and mobility score).

^c^ *P* < .05.

**Figure 2.**
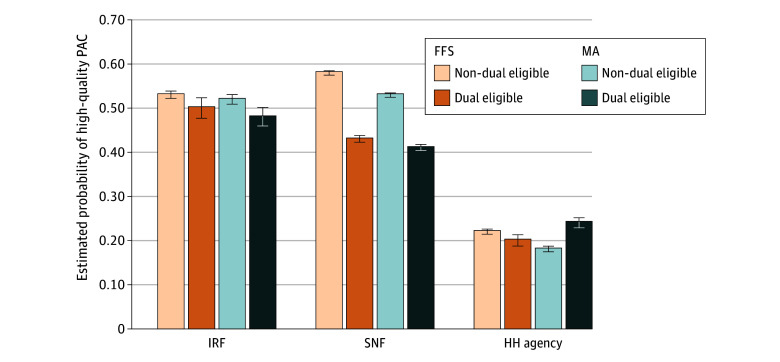
Estimated Probability of Receiving High-Quality Postacute Care (PAC) Across 4 Mutually Exclusive Groups Error bars indicate 95% CIs. FFS indicates fee-for-service Medicare; HH, home health; IRF, inpatient rehabilitation facility; MA, Medicare Advantage; and SNF, skilled nursing facility.

Figure 2 displays estimated probabilities across the 4 mutually exclusive groups. Maximum variability was observed for SNFs, with FFS non–dual-eligible patients having the highest estimated probability of discharge to a high-quality SNF (0.58; 95% CI, 0.57-0.58) and MA dual-eligible patients having the lowest probability (0.41; 95% CI, 0.40-0.41).

## Discussion

In this national cohort of Medicare beneficiaries hospitalized for stroke, we found significant disparities in discharge to high-quality PAC associated with MA enrollment and dual-eligibility status. Our intent was not to compare quality across the PAC settings (IRF vs SNF vs HH care), but to examine the differences within each type of the PAC settings. First, we found that MA patients with and without dual-eligibility status were more likely to be discharged to lower-quality SNFs, after accounting for patient demographics, stroke severity, functional status, and market-level factors. Second, these differences were pronounced among dual-eligible patients, regardless of their FFS or MA status, as demonstrated by a higher likelihood of admission to low-quality SNFs for those with FFS plans with dual eligibility. There were no significant differences in the quality of IRFs, but for HH care, MA non–dual-eligible patients were less likely to receive care from high-quality HH agencies. These findings underscore significant concerns regarding differential discharge to quality PAC settings for patients with stroke who are dually enrolled in Medicare and Medicaid and those with MA plans. These pronounced differences in discharge to high-quality SNF care may further worsen disparities in stroke recovery and long-term outcomes.

Our study extends the prior literature in 4 ways. First, we focus on the compounding association of dual-eligible status with MA plans on discharge to quality PAC settings. Our results show that patients with stroke are enrolling in different types of plans, with approximately 50% enrolling in MA plans. Dual-eligible patients with stroke were more likely to be enrolled in MA plans than FFS plans. We found that after adjusting for stroke severity, comorbidities, and functional status, in addition to hospital- and market-level factors, the probability of discharge to high-quality SNFs was lowest among dual-eligible individuals with MA plans. One of the unique features of the MA plans’ operation is the network size of their clinicians and facilities with whom a plan has a contractual agreement. Because of this, the MA plan may exert tighter control on improving efficiency and cost reduction.^13,14,44,45^ Inadvertently, if an MA plan has a network of all low-quality SNFs, then all patients covered under that plan would be discharged to those low-quality SNFs, as reported previously.^18,45,46^ In our study, dual-eligible individuals with MA plans could have been covered under such plans with a network of SNFs that were lower rated.^45,46^ It would be important to examine how such access differences are associated with the utilization of rehabilitation services at SNFs and other PAC settings, functional recovery, and long-term health outcomes.

Second, we found that a higher concentration of MA plans in a geographical region was associated with discharge to high-quality SNFs. The concentration of MA plans in a geographic area plays a crucial role in developing a mix of provider networks, shaping the quality of care received by beneficiaries. A higher number of MA plans also often leads to more competitive health care options, which can enhance PAC through improved provider networks and increased access to high-quality SNFs.^46^ Conversely, in areas with fewer MA plans, beneficiaries may face limited choices and potentially lower-quality care. Finally, due to variability in different stroke certifications for acute care hospitals (eg, primary stroke center, comprehensive stroke center), we opted to use stroke volume at acute care hospitals as a proxy to adjust for the quality of stroke care in the acute setting. We found that higher stroke volume was associated with discharge to a high-quality SNF, which could be indicative of an overlap between stroke volume and stroke certification, with the latter resulting in care coordination between the acute care hospital and (high-quality) SNF.

Third, to our knowledge, this is the first study to include the quality of IRFs, where we found no differences across the 4 groups. One major reason for this finding is that IRFs lack a validated star rating system, unlike SNFs and HH agencies, which have well-established and CMS-validated 5-star quality measures.^31,32,34^ Although these 5-star ratings are based on a composite measure of structure, process, and outcomes, IRFs do not have such measures that help capture quality. Thus, we had to rely on the use of individual quality measures that are part of the IRF Compare reporting. The quality measures that were used for IRFs are validated, endorsed by the National Quality Forum, and part of the IRF Compare.^36,37^ Testing the validity of using such a system is beyond the scope of this study; however, this could be one reason for the lack of sensitivity in the IRF quality rating.

Fourth, our study shows the importance of studying discharge to high-quality HH agencies. We found a significantly lower likelihood of access to high-quality HH agencies for non–dual-eligible individuals with MA plans. Prior evidence had reported discharge to lower-quality rating HH agencies for MA beneficiaries compared with FFS beneficiaries^17^ and lower utilization of HH services, resulting in lowered improvements in functional outcomes.^47^ For our study, there was a lower likelihood of access to high-quality HH agencies among regions with a high concentration of SNFs. Although the substitution for discharge between SNFs and HH care after hospitalization can be associated with reimbursement policies, and the differences between outcomes is documented in the literature,^48^ our results highlight the importance of examining the context of access to quality HH care, and how SNF supply is associated with access to quality HH care.

### Limitations

This study has some limitations. The associations described here are limited by the inability to distinguish between patients who did not receive any PAC and those receiving PAC services in outpatient (rehabilitation) settings, as we did not have access to outpatient (Medicare Part B) claims. We also did not adjust for types of MA plans and the heterogenous association for dual-eligible and non–dual-eligible individuals that might be associated with discharge to quality PAC. The missing information on stroke severity in claims data could have had residual confounding on care transition, from acute to PAC settings. However, we risk adjusted for patient comorbidities and functional status with measures from PAC settings at the patient level. We also acknowledge confounding by indication by condition severity, and baseline differences in both insurance and dual-eligibility status. The study findings need to be interpreted in the context of these limitations. Last, this work focused heavily on the descriptive nature of discharge to quality of PAC, and there was a lack of outcomes associated with access to high vs low quality of PAC. Despite these limitations, to our knowledge, this study is the first to address the compounding association of MA and dual-eligibility status in examining access to quality PAC settings for patients with stroke. This study uses information that has largely been overlooked in previous research, including standardized functional status and stroke severity. Although there are limitations to our study, we believe it contributes valuable insights to the literature.

## Conclusions

Our cohort study found significant disadvantages in discharge to quality SNFs for patients with stroke associated with dual eligibility and MA plans. These differences in quality of SNF care can be associated with health outcomes during the initial phases of stroke, particularly functional recovery, and prevention of infections, pressure ulcers, and hospital readmissions. MA plans may be incentivized to reduce costs by using SNFs that save money, even if those facilities deliver lower-quality care. With the concurrent growth of MA and dual enrollment, the design of value-based PAC for these populations is a priority to ensure they maximize gains from PAC services provision and achieve optimal health outcomes.

## Notes

Supplement 1. **eTable 1.** Adjusted Odds Ratio For Receiving High-Quality Post-Acute Care **eTable 2.** Main Effect of Medicare Plan Type and Dual-Eligible Status on Receiving High-Quality Post-Acute Care

Supplement 2.
Data Sharing Statement
