# Supplementary material for: Association of Dual Eligibility and Medicare Type With Quality of Postacute Care After Stroke
Source: JAMA Netw Open. 2026 Feb 24;9(2):e260095. doi: 10.1001/jamanetworkopen.2026.0095 (PMC12933277; doi:10.1001/jamanetworkopen.2026.0095)
Supplement: Supplement 1. — eTable 1. Adjusted Odds Ratio For Receiving High-Quality Post-Acute Care eTable 2. Main Effect of Medicare Plan Type and Dual-Eligible Status on Receiving High-Quality Post-Acute Care [file jamanetwopen-e260095-s001.pdf]

Supplementary Online Content

Karmarkar AM, Chou LN, Jain T, et al. Association of dual eligibility and Medicare type with quality of postacute care after stroke. *JAMA Netw Open.* 2026;9(2):e260095. doi:10.1001/jamanetworkopen.2026.0095

eTable 1. Adjusted Odds Ratio For Receiving High-Quality Post-Acute Care

eTable 2. Main Effect of Medicare Plan Type and Dual-Eligible Status on Receiving High-Quality Post-Acute Care

This supplementary material has been provided by the authors to give readers additional information about their work.

© 2026 Karmarkar AM et al. *JAMA Network Open.*

eTable 1. Adjusted Odds Ratio For Receiving High-Quality Post-Acute Care

Variable	Inpatient Rehabilitation Facility	Skilled Nursing Facility	Home Health Care
Number of Patients[&]	**16,180**	**10,137**	**10,283**
**Patient-Level**			
Insurance Plan			
FFS Non-Dual eligible (ref)	1.00	1.00	1.00
FFS Dual eligible	0.89 (0.70-1.14)	0.57 (0.50-0.65)*	0.79 (0.62-1.01)
MA Non-Dual eligible	0.98 (0.85-1.13)	0.82 (0.74-0.91)*	0.71 (0.62-0.82)*
MA Dual eligible	1.12 (0.89-1.40)	0.56 (0.50-0.64)*	0.91 (0.74-1.13)
Age, years	1.00 (0.99-1.01)	1.01 (1.01-1.02)*	0.99 (0.99-1.00)
Female	1.05 (0.93-1.18)	1.11 (1.02-1.20)*	0.92 (0.81-1.04)
Race/Ethnicity, n(%)			
African American/Black	1.03 (0.86-1.25)	0.79 (0.71-0.89)*	1.23 (1.02-1.48)*
Hispanics	1.37 (1.04-1.81)	0.72 (0.60-0.86)*	0.85 (0.65-1.13)
Non-Hispanic White	1.00	1.00	1.00
Others	1.11 (0.82-1.51)	0.97 (0.78-1.20)	0.79 (0.58-1.09)
Resident in Metropolitan	0.74 (0.60-0.91)*	1.04 (0.93-1.18)	0.84 (0.69-1.02)
Resident in Stroke Belt	0.90 (0.52-1.54)	0.70 (0.57-0.85)*	1.96 (1.37-2.79)*
Resident in High MA penetration	0.89 (0.72-1.09)	1.03 (0.91-1.16)	1.24 (1.02-1.52)*
Charlson Comorbidity Index	1.02 (0.98-1.07)	0.98 (0.96-1.01)	0.98 (0.94-1.03)
LOS in Acute Hospital, days	0.99 (0.97-1.01)	0.98 (0.97-0.99)	0.99 (0.97-1.01)
ICU Stay	1.01 (0.86-1.18)	1.11 (1.01-1.21)*	1.04 (0.90-1.22)
Self-Care at admission	1.02 (1.01-1.04)*	1.01 (1.00-1.01)	0.97 (0.96-0.99)*
Mobility at admission	1.00 (0.99-1.01)	1.00 (0.99-1.01)	1.00 (0.99-1.00)
NIH Stroke Severity			
NIHSS Score 0-9	1.00	1.00	1.00
NIHSS Score 10-42	1.11 (0.94-1.31)	0.88 (0.79-0.99)*	1.02 (0.80-1.29)
Unknown	0.94 (0.81-1.10)	0.83 (0.76-0.91)*	1.00 (0.87-1.15)
IV tPA during the inpatient stay	1.03 (0.84-1.27)	1.07 (0.92-1.24)	0.86 (0.68-1.08)
Hospital-based rehabilitation	5.03 (1.83-13.87)*	1.37 (0.95-1.96)	1.00 (0.64-1.58)
**Hospital-Level**			
**ICC (null model)**	0.6633	0.1214	0.2268
Major and Limited Teaching	0.85 (0.56-1.27)	1.06 (0.94-1.20)	0.86 (0.71-1.05)
High Stroke Volume	0.96 (0.63-1.45)	1.14 (1.01-1.29)*	0.96 (0.78-1.18)
**HRR-Level**			
**ICC (null model)**	0.1550	0.0901	0.2450
High Number of MA Plan	0.94 (0.52-1.71)	1.45 (1.17-1.81)*	1.10 (0.73-1.66)
High Number of IRF	1.09 (0.57-2.10)	1.02 (0.80-1.29)	1.18 (0.74-1.85)
High Number of SNF	1.42 (0.74-2.72)	0.91 (0.72-1.16)	0.56 (0.35-0.88)*
High Number of HHA	0.55 (0.29-1.05)	0.82 (0.64-1.04)	1.25 (0.79-1.97)

[*] $p<0.05$

[&] Only the subjects with complete data are included in the analyses

FFS: Fee-for-Service; MA: Medicare Advantage; IRF: Inpatient Rehabilitation Facility; SNF: Skilled Nursing Facility; HHA: Home Health Agency; MA: Medicare Advantage; LOS: Length of Stay; ICU: Intensive Care Unit; NIH: National Institutes of Health; NIHSS: National Institutes of Health Stroke Scale; tPA: Tissue plasminogen activator; ICC: Intraclass Correlation Coefficient

© 2026 Karmarkar AM et al. *JAMA Network Open.*

eTable 2. Main Effect of Medicare Plan Type and Dual-Eligible Status on Receiving High-Quality Post-Acute Care

**Variable**[&]	**Inpatient Rehabilitation Facility**	**Skilled Nursing Facility**	**Home Health Care**
**N**[&]	**16,180**	**10,137**	**10,283**
Patient-Level			
Medicare Plan			
FFS	1.00	1.00	1.00
MA	1.11 (0.94 - 1.31)	0.90 (0.82 - 0.98)*	0.90 (0.77 - 1.05)
Dual-Eligible			
Non-Dual	1.00	1.00	1.00
Dual	1.01 (0.85 - 1.20)	0.63 (0.57 - 0.69)*	1.00 (0.85 - 1.18)
Interaction, p-value	*p*=0.1562	*p*=0.0312*	*p*=0.0036*

[*] p<0.05

[&] Only the subjects with complete data are included in the analyses

Multilevel logistic regression model (HRR, Hospital and Individual) included the adjustment of patient-level covariates (age, sex, race, metropolitan, stroke belt location, MA penetration, Charlson comorbidity, acute hospital length of stay, ICU stay, National Institutes of Health Stroke Score (NIHSS), intravenous thrombolytic, inpatient rehabilitation, admission self-care score, and mobility score)

FFS: Fee-for-Service; MA: Medicare Advantage

© 2026 Karmarkar AM et al. *JAMA Network Open.*
