# Supplementary material for: Association of Dual Eligibility and Medicare Type With Quality of Postacute Care After Stroke
Source: JAMA Netw Open. 2026 Feb 24;9(2):e260095. doi: 10.1001/jamanetworkopen.2026.0095 (PMC12933277; doi:10.1001/jamanetworkopen.2026.0095)
Supplement: Supplement 2. — Data Sharing Statement [file jamanetwopen-e260095-s002.pdf]

Data Sharing Statement

Karmarkar. Association of Dual Eligibility and Medicare Type With Quality of Postacute Care After Stroke. *JAMA Netw Open*. Published February 24, 2026.
doi:10.1001/jamanetworkopen.2026.0095

Data
Data available: No
